# Supplementary material for: Modeling glioblastoma heterogeneity as a dynamic network of cell states
Source: Mol Syst Biol. 2021 Sep 16;17(9):e10105. doi: 10.15252/msb.202010105 (PMC8444284; doi:10.15252/msb.202010105)
Supplement: Supplementary file 6 — Source Data for Figure 5 [file MSB-17-e10105-s004.zip › Figure5A_sourcedata/GSEA_3071/hallmarks_stateII.GseaPreranked.1623742536692/HALLMARK_CHOLESTEROL_HOMEOSTASIS.html]

Details for gene set HALLMARK\_CHOLESTEROL\_HOMEOSTASIS[GSEA]

|  || Dataset | state23071 |
| Phenotype | NoPhenotypeAvailable |
| Upregulated in class | na\_neg |
| GeneSet | HALLMARK\_CHOLESTEROL\_HOMEOSTASIS |
| Enrichment Score (ES) | -0.24264647 |
| Normalized Enrichment Score (NES) | -0.9928983 |
| Nominal p-value | 0.4774775 |
| FDR q-value | 0.70654696 |
| FWER p-Value | 1.0 |
Table: GSEA Results Summary

  

Fig 1: Enrichment plot: HALLMARK\_CHOLESTEROL\_HOMEOSTASIS      
 Profile of the Running ES Score & Positions of GeneSet Members on the Rank Ordered List

  

| PROBE | GENE SYMBOL | GENE\_TITLE | RANK IN GENE LIST | RANK METRIC SCORE | RUNNING ES | CORE ENRICHMENT || 1 | PLAUR |  |  | 185 | 0.893 | 0.0103 | No |
| 2 | ALCAM |  |  | 288 | 0.771 | 0.0335 | No |
| 3 | EBP |  |  | 336 | 0.721 | 0.0669 | No |
| 4 | TMEM97 |  |  | 403 | 0.676 | 0.0930 | No |
| 5 | ACAT2 |  |  | 446 | 0.653 | 0.1234 | No |
| 6 | TNFRSF12A |  |  | 629 | 0.566 | 0.1140 | No |
| 7 | ACTG1 |  |  | 755 | 0.514 | 0.1155 | No |
| 8 | CYP51A1 |  |  | 952 | 0.458 | 0.0959 | No |
| 9 | PLSCR1 |  |  | 992 | 0.446 | 0.1142 | No |
| 10 | GPX8 |  |  | 1208 | 0.395 | 0.0861 | No |
| 11 | FDPS |  |  | 1348 | 0.367 | 0.0749 | No |
| 12 | ANTXR2 |  |  | 1791 | 0.299 | -0.0150 | No |
| 13 | ATF5 |  |  | 1794 | 0.299 | 0.0032 | No |
| 14 | NFIL3 |  |  | 2056 | 0.267 | -0.0443 | No |
| 15 | ERRFI1 |  |  | 2086 | 0.264 | -0.0349 | No |
| 16 | LDLR |  |  | 2128 | 0.260 | -0.0288 | No |
| 17 | ANXA5 |  |  | 2187 | 0.253 | -0.0272 | No |
| 18 | GNAI1 |  |  | 2254 | -0.254 | -0.0276 | No |
| 19 | TP53INP1 |  |  | 2337 | -0.263 | -0.0312 | No |
| 20 | FADS2 |  |  | 2338 | -0.263 | -0.0148 | No |
| 21 | ETHE1 |  |  | 2419 | -0.273 | -0.0174 | No |
| 22 | CPEB2 |  |  | 2787 | -0.333 | -0.0868 | No |
| 23 | ECH1 |  |  | 2885 | -0.353 | -0.0886 | No |
| 24 | FABP5 |  |  | 3513 | -0.549 | -0.2083 | Yes |
| 25 | CD9 |  |  | 3567 | -0.581 | -0.1850 | Yes |
| 26 | CTNNB1 |  |  | 3753 | -0.731 | -0.1849 | Yes |
| 27 | TM7SF2 |  |  | 3918 | -0.999 | -0.1627 | Yes |
| 28 | PNRC1 |  |  | 4022 | -1.397 | -0.1007 | Yes |
| 29 | CLU |  |  | 4069 | -1.908 | 0.0071 | Yes |
Table: GSEA details [plain text format]

  

Fig 2: HALLMARK\_CHOLESTEROL\_HOMEOSTASIS: Random ES distribution      
 Gene set null distribution of ES for **HALLMARK\_CHOLESTEROL\_HOMEOSTASIS**
